# A quantitative analysis of the mechanism that controls body size in *Manduca sexta*

**DOI:** 10.1186/jbiol43

**Published:** 2006-08-02

**Authors:** HF Nijhout, G Davidowitz, DA Roff

**Affiliations:** 1 Department of Biology, Duke University, Durham, NC 27708, USA; 2 Department of Ecology and Evolutionary Biology, University of Arizona, Tucson, AZ 85721, USA; 3 Department of Biology, University of California, Riverside, CA 92521, USA

## Abstract

**Background:**

Body size is controlled by mechanisms that terminate growth when the individual reaches a species-specific size. In insects, it is a pulse of ecdysone at the end of larval life that causes the larva to stop feeding and growing and initiate metamorphosis. Body size is a quantitative trait, so it is important that the problem of control of body size be analyzed quantitatively. The processes that control the timing of ecdysone secretion in larvae of the moth *Manduca sexta *are sufficiently well understood that they can be described in a rigorous manner.

**Results:**

We develop a quantitative description of the empirical data on body size determination that accurately predicts body size for diverse genetic strains. We show that body size is fully determined by three fundamental parameters: the growth rate, the critical weight (which signals the initiation of juvenile hormone breakdown), and the interval between the critical weight and the secretion of ecdysone. All three parameters are easily measured and differ between genetic strains and environmental conditions. The mathematical description we develop can be used to explain how variables such as growth rate, nutrition, and temperature affect body size.

**Conclusion:**

Our analysis shows that there is no single locus of control of body size, but that body size is a system property that depends on interactions among the underlying determinants of the three fundamental parameters. A deeper mechanistic understanding of body size will be obtained by research aimed at uncovering the molecular mechanisms that give these three parameters their particular quantitative values.

## Background

Body size is an obvious and important characteristic of animals. It is highly correlated with fitness, and an increase in body size is one of the most common trends seen in evolutionary biology. The mechanisms by which genes affect body size have been widely studied. Genetic dwarf and giant strains are known for many animals. In both vertebrates and invertebrates, the genes that affect body size commonly exert their effect by altering the production of growth factors, or by altering the cellular response to growth regulators [1,2]. There has been much recent interest in the developmental mechanisms that control body size in insects [3]. Much of this work has used *Drosophila *as a model system and has focused on elucidating the role of insulin signaling in the regulation of growth and size, and on discovering the degree to which genetically and environmentally induced changes in body size are associated with changes in cell size or cell number [3-9]. It is now well established that increased insulin signaling, through overexpression of insulin-like peptides or overexpression of the insulin receptor, results in increased body size, and that reduction in insulin signaling is accompanied by a reduction in body size. Although the empirical correlation between insulin signaling and body size is well documented in *Drosophila *and is believed to be widespread among insects, it is not at all clear by what mechanism insulin affects body size. Presumably insulin controls cytoplasmic growth and cell proliferation and this is directly related to somatic growth. But exactly how somatic growth is, in turn, related to the final body size that an individual achieves is a mystery. This gap in our understanding is in part due to the fact that in *Drosophila *we do not fully understand the chain of events that results in the termination of the growth phase when the larva has achieved its species-characteristic size [3].

Body size is also affected by nutrient quantity and quality [10,11]. Nutrient restriction causes a diminution of body size, and it appears that nutrients affect growth rate and body size primarily by altering the secretion of insulin-like peptides. Temperature also has an effect on body size, and higher temperatures generally result in the development of animals of smaller body size. The mechanism by which temperature produces this effect in *Drosophila *has not yet been elucidated, but it is understood in the larva of another insect, the moth *Manduca sexta*, commonly known as the tobacco hornworm [12].

Size determination depends critically on the mechanism that causes a larva to stop growing. In all insects, including *Drosophila*, the immediate stimulus for the cessation of growth is the secretion of ecdysone, so the mechanism that controls the secretion of ecdysone must be part of the mechanism that controls size. The chain of events that leads to the secretion of ecdysone in the context of size regulation is today best understood in *Manduca *[3,12,13], which has long served as the model organism for insect endocrinology and postembryonic developmental physiology [14]. The sequence of physiological events and feedback mechanisms that culminate in the cessation of growth in *Manduca *are now sufficiently well understood that they can be described in explicit quantitative terms.

Most biological regulatory systems are sufficiently complex and nonlinear that they cannot be credibly analyzed through standard thought experiments and control diagrams. A mathematical description, however, provides an objective method for establishing the level of our understanding of a process. This is because it forces us to be explicit about all underlying assumptions, and quickly reveals whether the hypothesized interactions can actually produce the observed behavior of a system. A mathematical description allows us to explore the way in which the genetic, physiological and environmental determinants of body size interact, and allows us to examine their relative significance in body-size determination under different genetic and environmental circumstances. An accurate and well supported mathematical description of a complex process can also be used to make predictions about how a system will behave under novel or extreme conditions, and to do *in silico *'experiments' that would be impractical or time-consuming to do on living organisms. We previously [12] proposed an endocrine-based physiological mechanism that describes the regulation of body size. In the two sections that follow we outline the critical features of that mechanism, which we then use to formulate a quantitative model for growth and size determination.

### Larval growth

In the laboratory, *M. sexta *has five larval instars, and thus undergoes four larval molts. The cuticle of most of the body wall of the larva is soft and pliable. During the intermolt period the soft cuticle grows in thickness, but also increases in surface area through intercalary insertion of the chitin and protein matrix that makes up the bulk of the cuticle [15]. Presumably this soft integument could grow indefinitely, and this should obviate the need for periodic molting, were it not for the heavily sclerotized head capsule and mouthparts, which cannot grow and can therefore only increase in size through a classical molting cycle. An additional constraint to intermolt growth is provided by the outermost layer of the cuticle, the epicuticle. The epicuticle is laid down as a finely corrugated sheet that gradually flattens out as the underlying cuticle grows [15,16]. The epicuticle is inextensible and once its fine folds are stretched flat it allows no further increase in the surface area of the cuticle.

Adult insects cannot grow and, therefore, adult body size is determined by the size the larva has reached when it stops feeding and begins the metamorphic molt. The final size of the larva, and as a consequence the size of the adult, is determined by three factors: the number of larval instars, the size increment at each larval molt, and the size within the last larval instar at which the larva stops feeding and initiates metamorphosis.

Evolution of body size could be accomplished by evolution in any one (or all) of these factors. In the insects there is a general phyletic trend to decrease the number of instars and increase the size increment at each larval molt [14,17]. The largest of insects, the Goliath beetles of Africa, only have three larval instars, so here evolution of large body size from a small-bodied scarabaeid ancestor (all of which have three larval instars) has clearly occurred by increasing the size increment at each larval molt. In the Lepidoptera too, to which *Manduca *belongs, the evolution of large body size is accompanied by an increase in size increment, not an increase in the number of larval instars.

The size increment at each molt (the growth ratio) is determined by the amount of cell division and cell enlargement of the epidermis that occurs at the time of the molt when the cuticle of the next instar is laid down. Evolution of the growth ratio is presumably due to evolutionary changes in the combined effects of cell multiplication and cell enlargement.

### The mechanism of body size determination in *Manduca*

The initial mass of each larval instar is a constant multiple of that of the previous instar (see below). This is a common feature of insect growth and is referred to as Dyar's rule. In *Manduca*, the last (fifth) larval instar grows from a mass of about 1.2 g to about 11 g, so almost 90% of the final mass of the larva is gained during this single instar. Because most of the body mass of *Manduca *accumulates during the last larval instar, variation in the mechanisms that control growth and size in the last larval instar will have a greater effect on final body size than variation in the mechanisms that operate in earlier instars.

**Figure 1:**
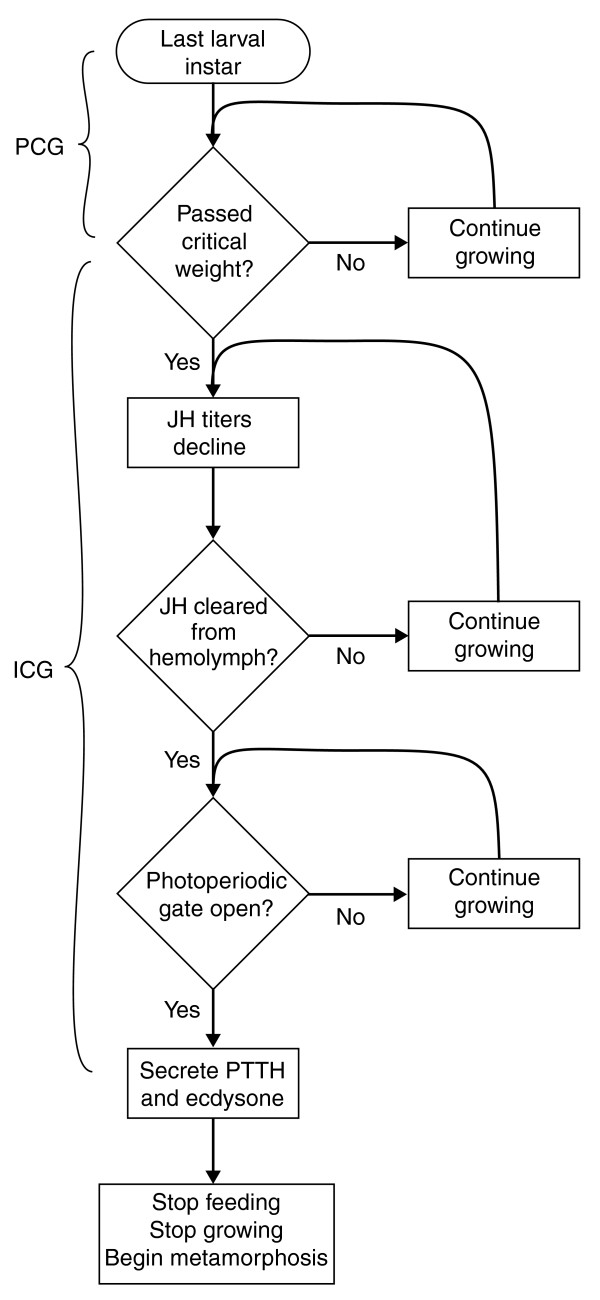
Flow chart for the mechanism that controls body size in *Manduca*. During the last larval instar there are three physiological decision points (diamonds) that control the timing of the cessation of growth. The amount of growth in the intervals between these conditional events determines the final size. PCG, growth prior to the critical weight; during this period the larva achieves approximately half its final mass. ICG, the interval to cessation of growth, which corresponds to the delay period between attainment of the critical weight and the secretion of prothoracicotropic hormone (PTTH) (see Figure 2).

It has been known for some time that the last larval instar has a distinctive developmental physiology that differs from that of the earlier instars [14]. In brief, in the last larval instar (but not in the earlier instars), the secretion of the prothoracicotropic hormone (PTTH) and the secretion of ecdysteroids are inhibited by juvenile hormone (JH). The titer of JH is high during the early portions of the instar, but in about the middle of the instar secretion of JH stops and the level of JH esterase (JHE), an enzyme that inactivates JH in the hemolymph, rises [18-22]. The titer of JH gradually declines, and when it has disappeared secretion of PTTH and ecdysteroids is disinhibited. The actual secretion of PTTH is controlled by a photoperiodic clock and can only occur during a well defined 'photoperiodic gate' [23,24], an 8 hour window of time that recurs each day. In *Manduca*, PTTH secretion occurs during the first photoperiodic gate after JH has disappeared from the hemolymph [14,25]. PTTH stimulates the secretion of ecdysteroids, and the initial rise of ecdysteroids causes the larva to stop feeding, purge its gut contents, and initiate a period of active 'wandering' in search of a suitable place to pupate. This decision pathway for size determination is illustrated in Figure 1.

**Figure 2:**
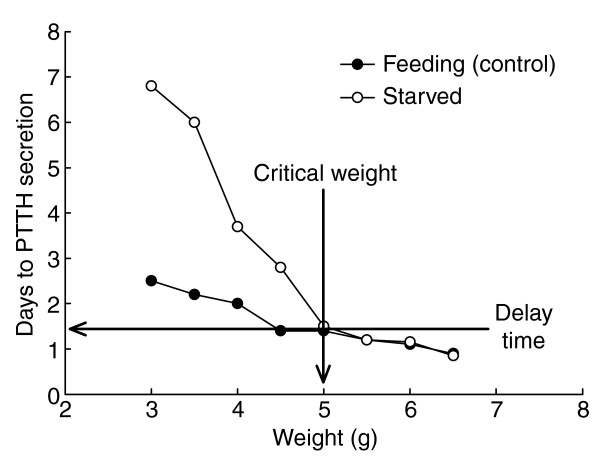
Method for establishing the critical weight and the delay time for PTTH secretion. Larvae are starved at various weights and the onset of wandering is determined and compared with that of larvae of similar weights that are allowed to continue feeding. The critical weight is the smallest weight at which there is no difference between starved and feeding larvae in the time required to initiate PTTH secretion and enter the wandering phase. The delay time is the time between achieving the critical weight and the actual secretion of PTTH, which corresponds to the ICG in Figure 1. Thus at the critical weight a series of events are set in motion that lead to PTTH secretion and that are not affected by subsequent nutrition. The critical weight occurs in about the middle of the growth phase of the fifth larval instar, so a larva can approximately double its mass after passing the critical weight.

The critical weight is the weight at which the secretion of JH stops, and this sets in motion a sequence of physiological events that culminate in the cessation of growth and the initiation of metamorphosis. Once JH secretion stops the subsequent series of physiological events is independent of further nutrition or growth. The critical weight can thus be operationally defined as the weight at which the time to the gut purge and initiation of wandering is independent of further feeding and growth (Figure 2). The value of this critical weight is determined by both genetic and environmental factors, and the evolution of body size in *Manduca *has been shown to be due, among other factors, to the evolution of the critical weight [13]. The mechanism by which a larva assesses its body size and its critical weight are not known [14], but the critical weight appears to be a function of the initial weight of the instar (see below).

The peak size a *Manduca *larva achieves in the final instar is thus a function of five variables: the initial size of the instar, the growth rate, the critical weight, the time required to break down JH, and the timing of the photoperiodic gate for PTTH secretion. There is genetic variation for each of these physiological determinants ([26] and our unpublished data); evolution of body size could therefore be due to evolutionary changes in one or more of these factors [13].

## Results

### Parameters for normal growth

**Figure 3:**
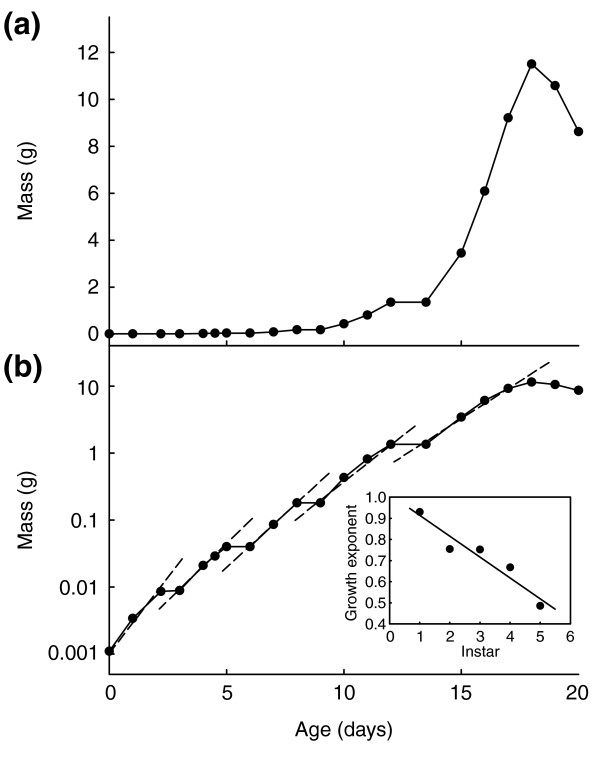
Growth trajectory of a typical larva. Growth occurs during five larval instars, separated by brief periods of molting during which no growth occurs. **(a) **Linear plot: the peak on day 18 is the maximum size the larva attained and marks the time at which the larva stopped feeding and growing. Decreasing mass after this time is due to the purge of gut contents. **(b) **Semilogarithmic plot: dashed lines are exponential regressions on the growth phases of each of the five larval instars. The inset shows a plot of the exponents of the regressions in (b) showing a linear decrease with instar (the regression is: exponent = 1.01 - 0.098*instar; *R*^2 ^= 0.92).

In our laboratory colony, larvae vary in their growth rate and in the peak size they attain before purging their gut. They purge on days 17 to 20 after hatching. The growth trajectory of a single representative larva of *Manduca *is shown in Figure 3. This individual purged its gut on the 18th day after hatching from the egg. Growth stops periodically as the larva molts from one instar to the next, and after day 18 the mass declines as the larva purges its gut contents, enters the wandering stage, and prepares for pupation. Overall growth during the feeding period is approximately exponential (Figure 3b), as is typical for insects. Within each instar, growth is also approximately exponential, but it is clear that the exponent declines progressively from instar to instar. Regression of the exponent value on instar number (Figure 3b inset) shows that the growth exponent declines approximately linearly from instar to instar. In any given instar the value exponent is given by: exponent = 1.01 - 0.098*instar. Thus, in the fifth instar the growth is described approximately by mass = *W*_0_*e^0.52*t^, where *t *is the time in days from the beginning of the fifth instar and *W*_0 _is the initial mass of the instar.

### Exponential size increase and the critical weight

**Figure 4:**
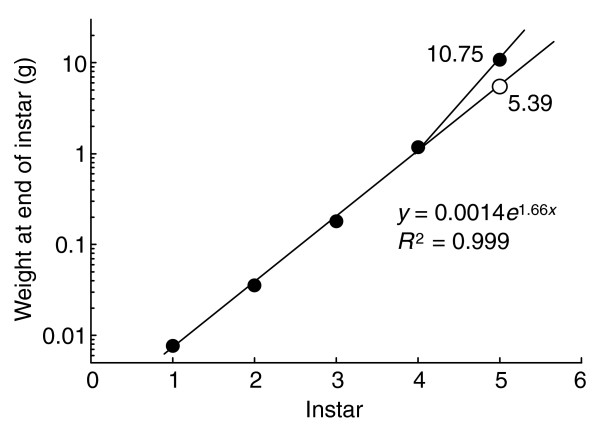
Final sizes of the five larval instars of *Manduca sexta*. The size of the first four instars increase exponentially, but the final size of the fifth instar is about twice (10.75 g) what would be expected (5.39 g) from the regression on the earlier instars. The regression is: weight = 0.0014*e^1.66*instar ^(*R*^2 ^= 0.999).

Although the growth exponent decreases gradually from instar to instar, the size increment from instar to instar is constant. That is, the final mass of each is a constant multiple of the final mass of the previous instar. As a consequence, the mass of a larva at each larval molt increases exponentially from instar to instar (Figure 4). The only exception to this rule is the final mass of the last larval instar, which is substantially larger than expected. Figure 4 illustrates that the predicted final mass of the fifth instar should be about 5.4 g, on the basis of a projection from an exponential regression on the masses of earlier instars. Thus, if *Manduca *had six or more larval instars, we would expect the fifth instar to molt to the sixth at a mass of about 5.4 g. The actual final mass of the fifth instar is about 11.5 g, about twice the expected mass.

**Figure 5:**
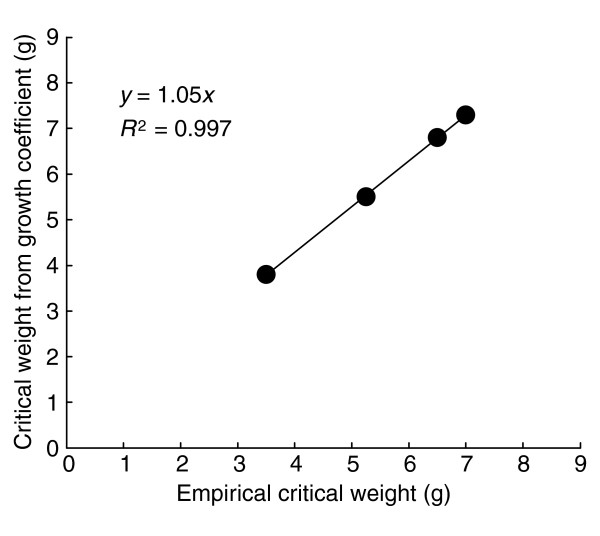
Relationship between the empirically measured critical weight of a fifth-instar larva and the predicted weight at which a fifth instar would have molted to a sixth larval instar. The predicted weight is based on the projected terminal weight of the last larval instar deduced from the exponential increase from instar to instar (Dyar's rule), as shown in Figure 4. The fit to a slope of 1 is excellent.

Interestingly, the predicted mass at a presumptive fifth to sixth instar molt (5.4 g) is very close to the critical weight (approximately 5.3 g) for the strain in which these measurements were made. We have also measured the critical weights and growth coefficients for several other genetic strains of *Manduca *that differ substantially in growth rate and body size (Figure 5). We found that there is an almost perfect linear relationship between the critical weight and the expected final mass of the fifth instar predicted by the size increments of earlier instars.

This finding suggests that the physiological changes initiated at the critical weight are somehow related to those that accompany a normal larval-larval molt. Moreover, this finding shows that the critical weight is a simple multiple of the mass of the larva at the beginning of the final larval instar.

It is therefore possible to derive an equation that relates the critical weight to the size increment and initial conditions. In general, the final size of each of the first four instars is a function of the size increment, and is given by the equation

  final mass of instar = *W*_1_*e^D*instar^,     (1)

where *W*_1 _is the mass of the hatchling larva and *D *is the size increment for the first four instars (= 1.66 in Figure 4). The critical weight (*CW*) in the fifth instar can also be estimated from the initial weight of the fifth larval instar (*W*_5_) as follows:

  *CW *= *W*_5_*e^*D*^.     (2)

In the several genetic strains of *Manduca *we have examined, *W*_5 _varies from 0.85 g to 2.25 g, and *D *varies from 1.45 g to 1.85 g. Genetic and environmental variation in the values of *W*_5 _and *D *will have profound effects on the value of the critical weight and, by extension, the final weight of the larva.

### Growth of the fifth larval instar

**Figure 6:**
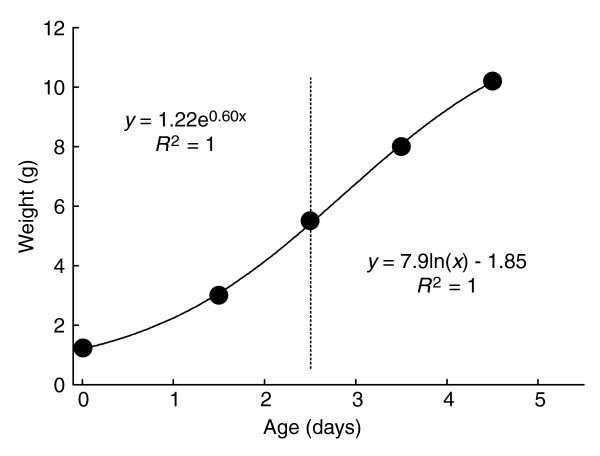
Growth of a fifth-instar larva with a critical weight of 5.2 g. The vertical dotted line is drawn through the time point at which the critical weight is passed. The growth trajectory before this time is concave upward and the trajectory after this time is concave downward, and the best-fitting equations for each of these segments of the growth trajectory are indicated.

A mean growth curve for a cohort of fifth-instar larvae from a laboratory colony of *Manduca *from ecdysis to the time of the gut purge is shown in Figure 6. Clearly, the overall growth during the fifth instar is not exponential but resembles a rather flat sigmoid. The slowdown and cessation of growth at the end of the instar are due to the secretion of ecdysteroids, which cause the larval to stop feeding and enter the wandering stage in preparation for pupation. The low growth rate at the beginning of the instar reflects the time necessary for the biochemical and physiological processes of molting to cease and those for feeding and growth to reactivate.

**Figure 7:**
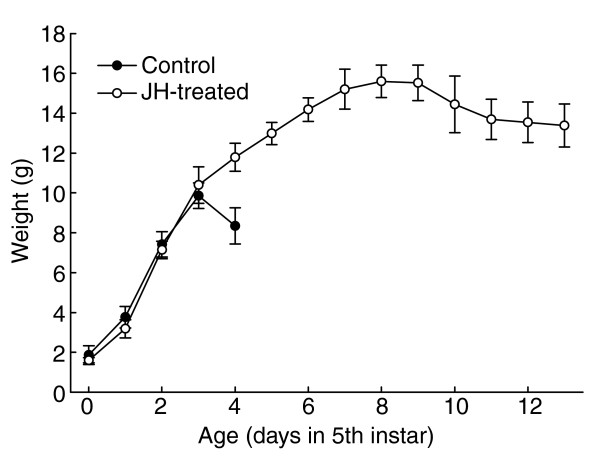
Growth trajectories of normal and JH-treated larvae of *Manduca*. JH-treated larvae (open circles) received a topical application of 50 μg methoprene (a stable JH analog) when they reached a weight of 3 g and again when they reached a weight of 6 g. Untreated larvae (filled circles) ceased feeding, purged their gut and entered the wandering stage on day 4. JH-treated larvae continued to feed for more than 2 weeks, but stopped growing after about 10–12 days, indicating that there is a physical limitation to the maximal size to which they can grow. Each curve is the mean of five larvae.

In order to derive an equation that describes growth during the fifth instar it is useful to know what the trajectory would look like in the absence of the influence of ecdysteroids, which can be thought of as prematurely terminating the growth phase. In the last larval instar, ecdysteroid secretion is inhibited by JH, and when larvae are treated with JH they continue to grow well beyond their normal final size [25,27]. It is therefore possible to deduce the shape of the uninterrupted growth trajectory by inhibiting the secretion of ecdysone with exogenous JH. When a topical application of 50 μg methoprene (a stable JH analog) is given on days 1 and 2 of the fifth instar, ecdysone secretion is inhibited and the larva continues to grow for at least a week beyond the time that growth would normally have stopped. The growth trajectory of JH-treated larvae is shown in Figure 7.

The overall growth curve of methoprene-treated larvae shows a gradually increasing growth rate until they reach a mass approximately equal to the critical weight (5.3 g for this strain of *Manduca*), followed by a decreasing growth rate above that weight. We assume that the integument poses an increasing resistance to growth as the larva increases in size, and that this accounts for the decreasing growth rate as the larva gets bigger. It is likely that growth in these larvae finally stopped at the maximal size allowed by the stretch of the epicuticle.

### The critical weight

The critical weight has an important role in controlling the final size of the larva. In the last larval instar, the critical weight marks the initiation of a dramatic change in physiology. After reaching the critical weight, the level of JHE in the hemolymph rises abruptly [21,22] and the JH titer gradually drops to zero. Once JH has disappeared, the secretion of PTTH and ecdysone are disinhibited. When ecdysone is secreted the larva stops feeding and growth stops.

**Figure 8:**
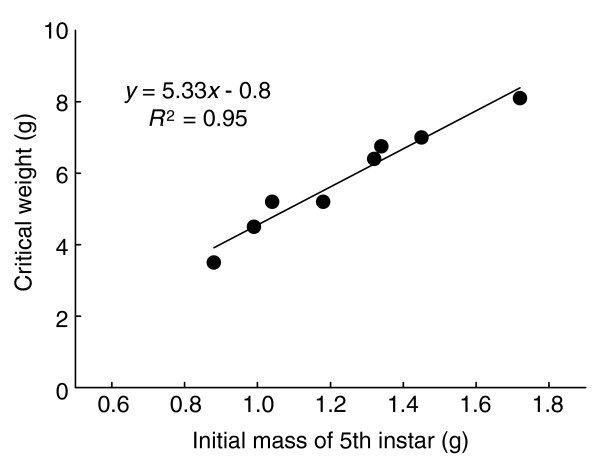
Relationship between the mass of the larva at the beginning of the last larval instar and the critical weight (*CW*), at which the decision to initiate the endocrine events that lead to metamorphosis is made. Each point is from a different genetic strain of *Manduca *that differs in body size and development time (G.D., D.A.R. and H.F.N., unpublished observations). The regression is: *CW *= 5.3*initial mass - 0.8 (*R*^2 ^= 0.95).

The mechanism by which a larva assesses its critical weight is unknown at present, but the data presented above show that it corresponds to the weight at which the fifth instar larva would have molted to the next larval instar, had it not been the final larval instar (see Figure 5). In addition, we have found that there is a simple linear relationship between the critical weight and the initial weight of the fifth instar larva across a broad range of body sizes and genetic backgrounds (Figure 8). The critical weights used to construct Figure 8 were determined using the method outlined in Figure 2, and show that the critical weight is approximately 5.3 times the initial weight of the instar, minus 0.8 g. This is in close accord with the interpretation of Figure 4. The critical weight thus has a simple linear relation to the initial weight of the final instar larva, and variation in the initial weight accounts for 95% of the variation in the critical weight (Figure 8).

There are various mechanisms that could have this property. What would be required is a measure or process that changes with the mass of the larva and for which the larva can measure the ratio between the current state and the state at the beginning of the instar. Stretch reception in which the length of the stretch receptor is set at the beginning of the instar provides a plausible mechanism [28,29], as does the prothoracic gland size measure described by [30].

### A mathematical description of growth and size determination

#### Growth before the critical weight

A model for growth and size determination must accurately replicate both the normal growth trajectory of a larva and the normal duration of the growth period. In other words, the model must account for both the growth trajectory and the decision point at which growth stops.

Growth is exponential until the critical weight is reached, after which the growth rate declines gradually. Exponential growth is described by

  d*W*/d*t *= k*W,     (3)

where *W *is the mass in g and *k *is the growth rate. Equation (3) has a solution:

  *W*(*t*) = *W*_5_*exp(k*t),     (4)

where *W*(*t*) is the mass at time *t*, *W*_5 _is initial weight at the beginning of the fifth instar, and *k *is the growth exponent. The growth exponent can be deduced from the size of the larva at a given time by solving equation (4) for *k*:

  *k *= ln(*W*(*t*)/*W*_5_)/*t*.     (5)

**Figure 9:**
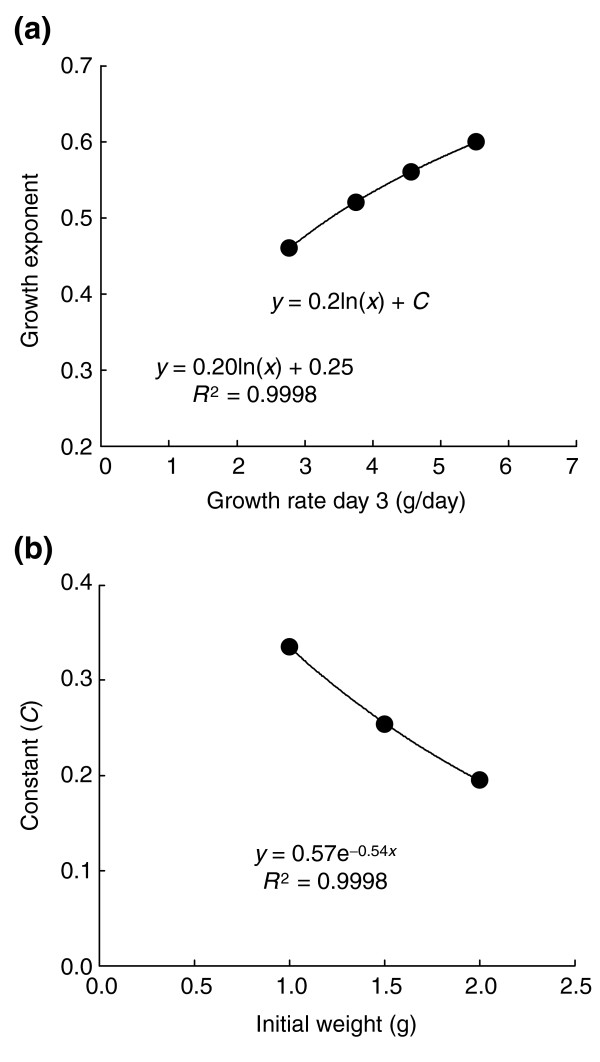
Derivation of growth exponent from growth rate on day 3. **(a) **The relationship between growth rate and growth exponent for larvae with the same initial weight. The relationship is best fit by a logarithmic regression where *k *= 0.2*ln(*GR*) + *C*, where *C *is a constant that depends on the initial weight of the instar. In this regression the initial weight was 1.5 g, which gives *C *= 0.25. **(b) **The relationship between *C *and the initial weight. An exponential regression gives the best fit. Substituting the equation in (b) for the constant *C *in *k *= 0.2*ln(*GR*) + *C *gives *k *= 0.2*ln(*GR*) + . So *k *can be deduced from the initial weight of the instar and the growth rate on the third day of the instar.

Because the overall growth curve is a rather flat sigmoid with the inflection point at around the critical weight (on day 3 under our 'standard' conditions), the growth rate between days 2 and 4 of the fifth instar larva is approximately linear. We have found that a close approximation of the growth exponent, *k*, can be obtained from the growth rate on day 3 and the initial weight of the instar. This eliminates the need to obtain a long series of weight measurements to determine the value of *k*. We begin by establishing the relationship between the growth exponent (*k*) and the growth rate (*GR*) on day 3 (Figure 9a). The best fit to this relationship is given by *k *= 0.2*ln(*GR*) + *C*, where *C *is a constant that depends on the initial weight of the fifth instar larva, which as shown in Figure 9b is given by *C *= . Combining these two equations gives:

  *k *= 0.2*ln(*GR*) + .     (6)

Equations (3) and (4) thus describe growth until the critical weight is achieved. The time at which the critical weight is reached (*t*_*CW*_) can be obtained by setting the left-hand side of equation (4) to the value of the critical weight and solving for *t*, which gives:

  *t*_*cw *_= ln(*CW*/*W*_5_)/*k *    (7)

#### Growth after the critical weight

**Figure 10:**
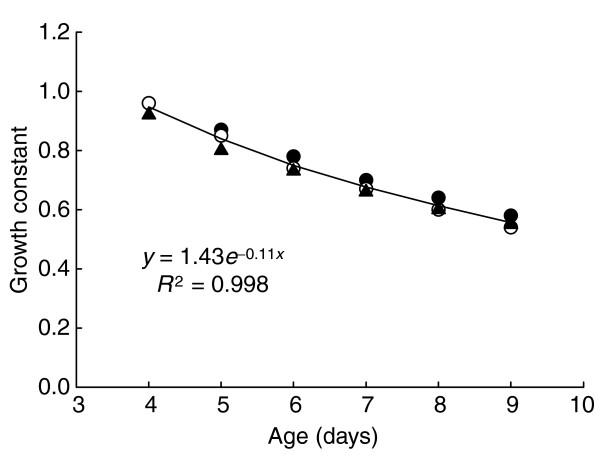
Variation in growth rate constant. Empirical growth data for three different strains: H (filled circles), B (triangles) and D (open circles), shows that all have the same rate of decay of the growth constant.

As noted above, the critical weight also marks the point at which the growth rate of the last instar larva changes from exponentially increasing to gradually declining (see Figure 6). This implies that the growth exponent must decline as the larva grows past its critical weight. The rate of decline of the growth exponent can be derived empirically from the growth trajectories of JH-treated larvae (Figure 10). We found that the rate of this decline is the same in larvae with different growth rates and different maximal sizes, and we assume, therefore, that it is characteristic of the species, rather than of a particular individual or genetic strain. After the critical weight, the growth of a larva is therefore given by

  d*W*/d*t *= k*d*W,     (8)

where *d *describes the rate of decline of the exponent *k*. The analysis in Figure 10 shows that *d *= 1.43*exp(-0.11*t). Substituting this formula for *d *into equation (8) and solving the differential equation gives the following expression for growth after the critical weight:



where *CW *is the critical weight and t is the time in days.

#### Duration of the growth period

In our strains of *Manduca*, ecdysis to the fifth instar occurs between 2 hours and 6 hours after the lights are switched on, on a cycle of 16 hours light and 8 hours dark (a 16L:8D photoperiod), and feeding begins within an hour after ecdysis. So, for the purposes of the model, we assume that a larva begins to grow 4 hours after lights-on. We define a day as the interval between lights-off signals, and designate the day on which growth begins as day 0 (zero).

The growth period ends with the secretion of PTTH and ecdysone. During this period growth is partitioned between the pre- and post-critical weight growth. The duration of pre-critical-weight growth is given by Equation (7). The duration of post-critical weight growth is determined by the mechanism that controls PTTH secretion. PTTH secretion can occur only during a well defined photoperiodic gate, and in fact occurs during the first photoperiodic gate after JH disappears from the hemolymph [14,25]. The mean time required for these processes differs in different genetic strains and must be determined by means of a critical weight experiment, as outlined in Figure 2.

The photoperiodic gate for PTTH secretion is between 14 hours and 24 hours after lights-off [11]. Thus the interval between the closing of a photoperiodic gate and the opening of the next one is about 16 hours. A larva that becomes competent to secrete PTTH just before a gate closes will do so, but if a larva becomes competent to secrete PTTH just after a gate closes it will continue to grow for an additional 16 hours, during which it can add an additional 1–2 g of weight (depending on its growth rate).

#### Parameters of the model

The overall model thus consists of Equations (3) and (8) and requires two parameters that relate to size and growth: the initial mass of the fifth larval instar, *W*_5 _(or the critical weight, *CW*, which is a simple linear function of *W*_5_), and the growth rate on day 3, *GR *(or the growth exponent, *k*, which is related to *GR *as shown in Equation (6)). The model also requires four parameters that relate to time: the time at which growth starts, the mean time interval between achieving the critical weight and PTTH secretion (called the interval to cessation of growth, or ICG [12]), and the opening and closing times of the photoperiodic gate. All these parameters are empirically measurable and should be characteristic of a given genetic strain. Changes in only three of these parameters (GR, ICG and CW) have been shown to fully account for the evolution of body size in a laboratory strain of *Manduca *[13].

The model can be run on a computer by numerical integration of Equations (3) and (8), using time steps of one half hour (or less) and keeping track of the time at which the critical weight is attained (at which time Equation (8) replaces Equation (3)), and the time at which photoperiodic gates open and close. Alternatively, the model can be run by calculating the time for the critical weight and the time of opening of the first gate after the ICG and substituting these values into Equations (4) and (9).

### Tests and predictions of the models

**Figure 11:**
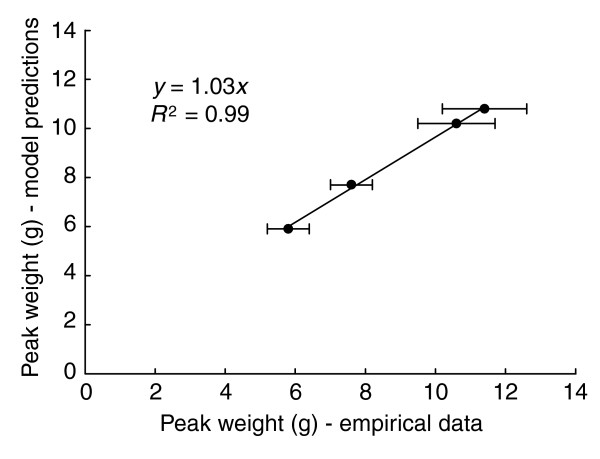
Predicted body sizes. Model predictions of peak weight of larvae of four different genetic strains of *Manduca *that differ in their growth parameters. 'Empirical data' are from Table 1. Bars are standard deviations.

Table 1 shows the parameter values and actual peak weights of larvae of four different strains of *Manduca*, and Figure 11 shows the relationship between the actual peak weights of these strains and their predicted peak weights using these parameter values. The model produces excellent predictions of the sizes of genetic strains with different growth parameters.

**Figure 12:**
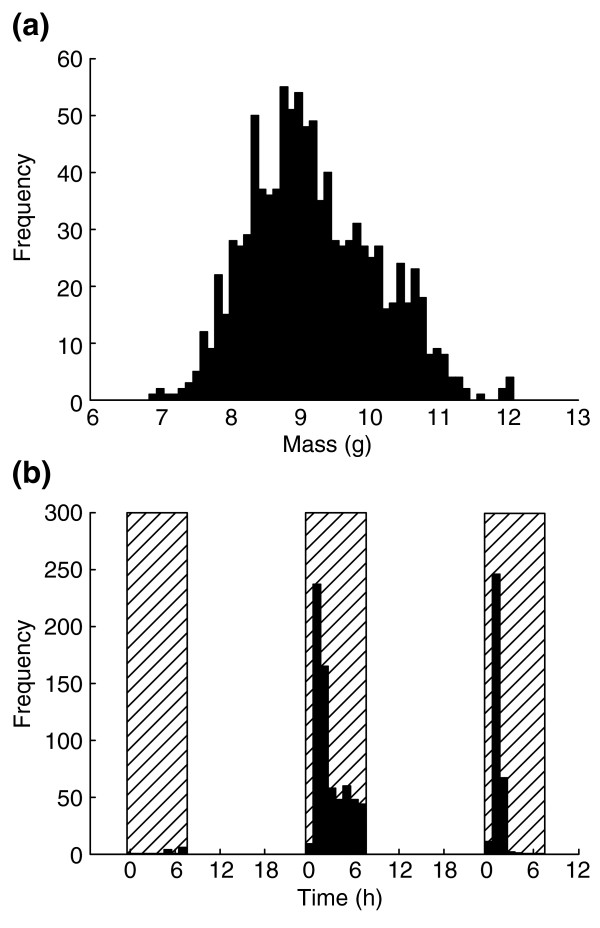
Simulation of population variation in body size and development time. One thousand individuals were generated with small amounts of random variation in all parameter values of the model. **(a) **Frequency distribution of peak sizes; **(b) **frequency distribution of times at which peak size was reached and wandering stage began. Hatched areas are photoperiodic gates.

In real life no two larvae will have exactly the same parameter values for the determinants of body size, because these are affected by both genetic and environmental variation. We therefore examined the effect of introducing variation in each of the parameters, by allowing them to vary randomly with a mean given by the parameter values for the H strain (Table 1) and a standard deviation (arbitrarily chosen) of 8% of the mean. Under these conditions, the peak mass of the larvae is approximately normally distributed, but the time required to reach the peak mass is multimodal (Figure 12). This is because the photoperiodic gating of PTTH secretion leads to a periodic distribution of the duration of the growth period. Interestingly, this has no appreciable effect on the size frequency distribution. A few animals reach their peak weight on the fourth day of growth, the majority do so on the fifth day, and the remainder on the sixth day. In each case a so-called 'gating bias' [23] is evident: the first larvae to reach peak weight do so relatively late in the gate, and for the subsequent days the majority of larvae peak early in the gate. The reason for this is that if larvae become competent to secrete PTTH while the gate is closed, they have to 'wait' until the next gate opens, and will thus release PTTH and achieve their peak weight very soon after the next gate opens. In this simulation, most of the individuals in the last group of larvae evidently became competent sometime during day 6 and therefore stopped growing (and thus reached their peak weight) shortly after the gate opened.

**Figure 13:**
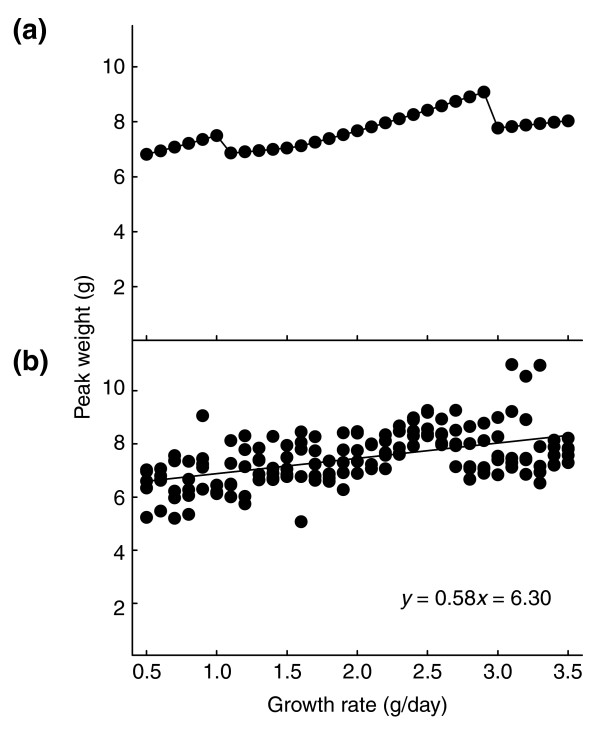
Model predictions of the effect of growth rate on body size. **(a) **Predicted effect of variation in growth rate on peak size; **(b) **predicted effect of variation in growth rate on peak size in the presence of a small amount of random variation in all other parameter values. Variation in the generating parameters masks the sawtooth character of the 'ideal' relationship. The line is a linear regression.

Variation in food quality alters the growth rate without affecting other parameters (G.D. and H.F.N., unpublished results); how does it affect peak weight? The relationship between growth rate and peak weight is illustrated in Figure 13a. The 'sawtooth' character of this relationship is due to the gating of PTTH secretion. As the growth rate increases, the time of PTTH secretion occurs progressively earlier in a gate, until the beginning of that gate is reached after which all larvae secrete PTTH at the beginning of the gate; then as growth rate increases further there is an abrupt switch to the gate of the previous day. Peak weight increases gradually with growth rate but drops abruptly when larvae shift to an earlier gate, after which the gradual increase continues. This kind of sawtooth relationship is seen in experimental data on larvae that vary in growth rate (G.D., unpublished results).

As before, in real larvae all the other parameters of size regulation vary among individuals, so in real life we should not necessarily expect to observe the idealized relationships shown in Figure 13a. Imposing normal random variation (with standard deviations 8% of means, as before) on the other parameters, using the T strain parameter values (from Table 1), while varying growth rate systematically gives the relationships shown in Figure 13b. Linear regression on the simulated results in Figure 13b gives the following relationship between growth rate and peak weight: peakweight = growthrate*0.58 + 6.31. This is close to the empirically observed relationship for this strain: peakweight = growthrate*0.56 + 6.30. Using the H strain parameter values we obtain the predicted relationship peakweight = growthrate*0.90 + 8.32, whereas the empirical relationship is peakweight = growthrate*1.03 + 8.23. Using the B strain parameter values the model predicts the relationship peakweight = growthrate*0.78 + 5.05, and the empirical relationship is peakweight = growthrate*1.07 + 4.19. Thus, the model predicts the correct slope and intercepts of the linear relationship between growth rate and peak weight very accurately for the T and H strains, but not as accurately for the B strain.

### Body size and development time

**Figure 14:**
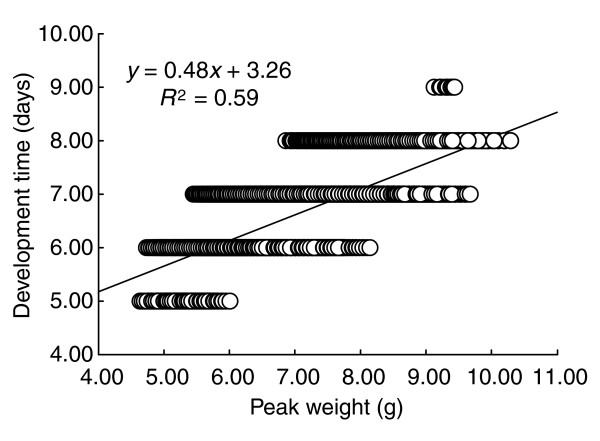
Relationship between body size and development time. Variation was introduced by allowing growth rate, critical weight and the ICG to vary with a standard deviation of 10% of the mean values of strain B in Table 1. The line is a linear regression on the data from 20,000 individuals (circles, many of which overlap in this plot).

The equations for the determination of body size are time-dependent and therefore they also embody the relationship between body size and development time (here we assume development time to be equivalent to the duration of the fifth larval instar). Development time and peak weight interact in a complex way because development time is determined, in part, by the time at which the critical weight is reached [12], which depends on the growth rate; and the growth rate also determines the amount of mass that is added after the critical weight is passed. Figure 14 shows the relationship between peak weight and development time under variation in the three fundamental parameters.

### Covariance between body size and the components of the mechanism

The mathematical model we have developed can be used to predict how variation in the three fundamental determinants of body size should affect variation of body size. To do this we need to find the functional relationship between peak weight and each of these three parameters. Because the effect of each determinant on peak weight size is nonlinear, and because the determinants interact with each other nonlinearly, there is no unique relationship between variation in any one of them and the peak weight size.

**Figure 15:**
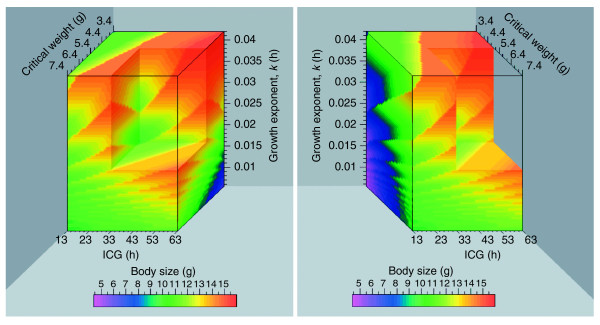
Body size as a simultaneous function of the three fundamental parameters. The three parameters describe a volume of parameter space in which body size is depicted on a color scale. The two panels show different views of the same graph. The cutout is made to illustrate some of the data within the volume. The sawtooth-like discontinuities arise from the photoperiodic gating of PTTH and ecdysone secretion (see Figure 12a for a one-dimensional representation). ICG, the interval to cessation of growth.

The relationship between any given parameter and peak weight depends on the specific values at which the other parameters are held constant. Therefore, body size cannot be expressed as a simple mathematical function of the three fundamental parameters, but the relationship between body size and each parameter must be found by solution or numerical simulation of the generative equations. It is possible to compute the peak weight that corresponds to any combination of values of the three fundamental parameters (as was done in Figures 12, 13, 14). The three parameters can be used as the orthogonal axes of a three-dimensional volume in which each location gives the body size for a specific triplet of parameter values. Such a volume is illustrated in Figure 15. Such a graphical representation illustrates the complexity and context dependency of the relationship between any given parameter and body size.

## Discussion and conclusions

### Three fundamental parameters

Body size in insects is determined by the mechanism that controls the secretion of ecdysone at the end of larval life. Ecdysone secretion causes the larva to stop feeding and prepare for metamorphosis. No further growth is possible and the size the larva has achieved at the time ecdysone is secreted fully determines the body size of the adult. Our quantitative analysis of the processes that lead to the secretion of ecdysone produced a simple mathematical model that predicts the correct body size and the correct relationship between growth rate and body size for diverse genetic strains of *Manduca *over a broad range of parameter values.

Although the mechanism that controls the secretion of ecdysone is complex and has many steps, its properties are determined by two parameters that relate to size and growth (the initial weight or the critical weight, and the growth rate), and three parameters that relate to time. These are: the time required to eliminate JH and derepress PTTH and ecdysone secretion (the ICG); and the times of opening and closing of the photoperiodic gate for PTTH secretion. Of these, the photoperiodic gate appears to be identical for all strains we have examined so far (G.D., D.A.R. and H.F.N., unpublished results), which means that only three fundamental parameters control variation in body size in response to genetic and environmental variation: the growth rate, the critical weight and the ICG. These three parameters are the same ones that were shown to be responsible for evolutionary changes in body size [13] and for phenotypic plasticity of body size [11] in a laboratory colony of *Manduca*.

Our analysis has given new insight into the properties of the critical weight. Until now, the critical weight was known only as an empirical measure of the point at which development becomes independent of nutrition, a point that corresponds to the time at which JHE in the hemolymph rises and JH is cleared. Our analysis shows that there is a simple linear relation between the critical weight and the initial weight of the instar, showing that the critical weight is a relative measure that depends on the prior growth history of the larva and the growth increment at each molt, which determine the initial size of the final larval instar. Moreover, the critical weight corresponds to the weight at which a larva would have molted to the next instar (had it not been in the final instar), which indicates that there is an as yet undiscovered regulatory mechanism that determines the size at which a larva-to-larva molt will occur, and that this mechanism is somehow related to the critical weight.

### The control of body size

So what 'controls' body size? Many investigators working at the molecular level have argued that insulin signaling is somehow in control, because if insulin signaling is disrupted, size regulation goes awry. It is clear from the structure of the mechanism we describe here, however, that insulin must have some intermediate role within one of the components of the mechanism, because we did not need to account for insulin explicitly. It is likely that insulin has a critical role in stimulating cellular growth and proliferation, and its function is therefore probably part of the growth-rate parameter in our mechanism. Insulin signaling also affects the synthesis of JH in *Drosophila *[31] and may therefore have an effect on body size regulation via this mechanism as well. The growth rate has a strong influence on body size (Figure 13, and see Equations (4) and (9)) because it affects how much mass accumulates between the critical weight and the secretion of PTTH and ecdysone. During this period the larva can more than double its mass, depending on its growth rate. In discussing Figure 13 we assumed that variation in growth rate was due to variation in nutrient intake, and insofar as nutrients exert their cellular effect by modulating insulin signaling, variation in the growth rate can be considered functionally equivalent to variation in insulin signaling. Of course there are many other variables that affect the growth rate, such as temperature and the availability of micronutrients and vitamins, and all these factors must interact in some way. It should therefore eventually be possible to write a more detailed mathematical description of the growth rate parameter that takes all these molecular and cellular interactions into account. In the meantime we can account for the effects of insulin signaling alone by assuming a specific (for example linear) relationship between insulin signaling and growth rate.

It should be clear that there is no single locus of 'control' of body size. In a complex mechanism it is possible to disrupt any number of components and affect the outcome, but that should not be construed to imply that one of those components somehow controls the outcome. Body size is a system property and results from the interplay of many equally important components. In this case we identified three fundamental parameters. Each of these parameters, in turn, is a complex system with many sub-components and long causal chains of interactions that establish their particular value in any one individual. It should therefore eventually be possible to develop a more detailed model with mathematical expressions for the growth rate as a function of nutrition and insulin signaling, and of the ICG as a function of JH synthesis, sequestration and catabolism.

### Plasticity of body size

We can now give a mechanistic interpretation to our previous results [11], which showed that plasticity of body size in response to diet quality is due to variation in growth rate and critical weight, whereas plasticity of size in response to temperature is due to variation in growth rate and the ICG. The effect of diet quality is manifested as variation in the growth rate, as noted above. In earlier instars the growth rate affects the size at which the larva molts, and larvae feeding on a poor-quality diet molt to each instar at a slightly smaller size than larvae feeding on a better diet [32]. Hence the size of the larva at the outset of the fifth instar will be affected by diet quality, and this, in turn, affects the critical weight.

Temperature has a direct effect on the rate of biochemical reactions. High temperature increases the growth rate and also increases the rate at which JH decays during the ICG, and this shortens the ICG. In insects there is an inverse relationship between body size and environmental temperature [33], and in *Manduca *this relationship is explained by the interaction between the effects of temperature on growth rate and the length of the ICG [12].

### Evolution of body size

Because the mathematical model defines the relationships between the underlying developmental parameters and final body size, it can be used to predict how the fundamental parameters should change under selection on body size. Figure 15 represents a three-dimensional phenotypic landscape [34,35] for body size. The mean and variance of a population can be depicted as a volume within the parameter space of Figure 15 and the gradient along each parameter axis can be calculated. Given a specific assumption about the nature of genetic variability for each parameter, evolution on such a landscape can be calculated using the methods outlined in [35].

### Broader applicability of the model

It is worth considering whether the mechanism we have uncovered in *Manduca *is applicable to other insects. Various authors, working mainly with *Drosophila*, have suggested that the control of body size resides at the level of insulin and signaling through the pathway involving phosphoinositide 3-kinase and target of rapamycin (TOR) [2,9,36], activity of the small GTPase Ras in the prothoracic glands [37], an antagonistic action between insulin and ecdysone [8], and the relative size of the prothoracic gland [30]; and that the control of body size must somehow depend on mechanisms that regulate cell size or cell number [38]. Most of this work was done using artificial genetic constructs that disrupt or enhance specific molecular pathways in specific tissues, and it has therefore been difficult to deduce how these proposed mechanisms interact and exactly how they play out in the normal regulation of growth and body size. Surely these molecular events are important parts of the complex network of interactions that establish final body size, but it is difficult to see how they can be in 'control' in the traditional meaning of the term. As noted above, body size regulation is a system property and in order to understand the system it is unhelpful to assume that control resides at any one point in the nexus of interactions.

With the exception of the recent work of Mirth *et al*. [30], previous studies on *Drosophila *have described growth and body size in qualitative, not quantitative terms. Hence it has been difficult to deduce whether the mechanism of size regulation in *Drosophila *and *Manduca *have anything in common. By carefully quantifying growth and body size, Mirth *et al*. [30] were able to show that *Drosophila *has a critical weight that is physiologically similar to that of *Manduca *and the mechanism we describe here for body size regulation in *Manduca *may therefore apply to *Drosophila *as well. The principal difference is that starvation in *Drosophila *can accelerate the onset of the PTTH and ecdysone secretion and the wandering phase. This is likely to be an adaptation to food exhaustion in a species that has little or no ability to find a new food resource, as has been described in the beetle *Onthophagus *[39]. This effect of starvation can be quantified and can be used to develop a *Drosophila*-specific variant of our model for size regulation.

## Materials and methods

We focused on events in the last larval instar because 90% of the increase in mass occurs during this developmental stage, and because all the processes that affect final body size occur during the last larval instar. We derived differential equations that describe growth under normal and experimental conditions and, wherever possible, solved these equations so that they expressed the various features of the mechanisms of growth and size determination as functions of the fundamental underlying variables. We tested the resulting mathematical description by examining whether it accurately describes growth and size regulation under various aberrant or extreme genetic and environmental conditions that were not considered in developing the model. Numerical simulation of the mathematical model was done using Matlab (The Mathworks).

Several strains of *Manduca *were used to establish the parameter values for growth and size regulation. Unless otherwise mentioned, most of the growth data were obtained from the wild-type strain obtained by hybridizing laboratory strains obtained from University of Washington, University of Arizona and North Carolina State University, designated strain H. Other strains used were the black larval strain, a recessive mutation in the JH-regulatory pathway that approximately halves body size, designated strain B; a strain produced by selection for large body size and long development time, designated strain D; and a strain that had been recently collected from the wild, whose parameters for growth and size regulation were originally measured in 1972 (see [13]) and which is designated here as strain W.

## Figures and Tables

**Table 1:** Parameter values for four genetic strains of *Manduca *used to generate model results shown in Figure 11

	Strain			
	
Parameter	B	W	H	D
*W*_0 _(g)	0.9	1.25	1.3	1.45
*CW *(g)	3.5	5.2	6.4	7.0
ICG	1.25	1.0	1.5	1.5
*GR *(g/day)	1.5	2.2	2.4	2.4
*W*(*t*)	3.4	4.7	5.5	6.1
*t *(days)	2.5	2.5	2.5	2.5
Peak weight (g)	5.9	7.7	10.2	10.8
SD	0.6	0.6	1.1	1.2

The average peak weight and standard deviation (SD) for each strain are shown. *W*_0_, initial weight of fifth instar larvae; *CW*, critical weight; ICG, interval to cessation of growth, that is, the interval between attainment of the critical weight and the secretion of PTTH; *GR*, growth rate; *W*(*t*), weight at time *t *(2.5 days in all cases reported here).
